# Nearshore Pelagic Microbial Community Abundance Affects Recruitment Success of Giant Kelp, *Macrocystis pyrifera*

**DOI:** 10.3389/fmicb.2016.01800

**Published:** 2016-11-14

**Authors:** Megan M. Morris, John M. Haggerty, Bhavya N. Papudeshi, Alejandro A. Vega, Matthew S. Edwards, Elizabeth A. Dinsdale

**Affiliations:** ^1^Department of Biology, San Diego State UniversitySan Diego, CA, USA; ^2^Bioinformatics and Medical Informatics, San Diego State UniversitySan Diego, CA, USA

**Keywords:** gametophytes, kelp recruitment, *Macrocystis pyrifera*, microbial ecology, macroalgae–microbial interaction

## Abstract

Marine microbes mediate key ecological processes in kelp forest ecosystems and interact with macroalgae. Pelagic and biofilm-associated microbes interact with macroalgal propagules at multiple stages of recruitment, yet these interactions have not been described for *Macrocystis pyrifera*. Here we investigate the influence of microbes from coastal environments on recruitment of giant kelp, *M. pyrifera*. Through repeated laboratory experiments, we tested the effects of altered pelagic microbial abundance on the settlement and development of the microscopic propagules of *M. pyrifera* during recruitment. *M. pyrifera* zoospores were reared in laboratory microcosms exposed to environmental microbial communities from seawater during the complete haploid stages of the kelp recruitment cycle, including zoospore release, followed by zoospore settlement, to gametophyte germination and development. We altered the microbial abundance states differentially in three independent experiments with repeated trials, where microbes were (a) present or absent in seawater, (b) altered in community composition, and (c) altered in abundance. Within the third experiment, we also tested the effect of nearshore versus offshore microbial communities on the macroalgal propagules. Distinct pelagic microbial communities were collected from two southern California temperate environments reflecting contrasting intensity of human influence, the nearshore Point Loma kelp forest and the offshore Santa Catalina Island kelp forest. The Point Loma kelp forest is a high impacted coastal region adjacent to the populous San Diego Bay; whereas the kelp forest at Catalina Island is a low impacted region of the Channel Islands, 40 km offshore the southern California coast, and is adjacent to a marine protected area. Kelp gametophytes reared with nearshore Point Loma microbes showed lower survival, growth, and deteriorated morphology compared to gametophytes with the offshore Catalina Island microbial community, and these effects were magnified under high microbial abundances. Reducing abundance of Point Loma microbes restored *M. pyrifera* propagule success. Yet an intermediate microbial abundance was optimal for kelp propagules reared with Catalina Island microbes, suggesting that microbes also have a beneficial influence on kelp. Our study shows that pelagic microbes from nearshore and offshore environments are differentially influencing kelp propagule success, which has significant implications for kelp recruitment and kelp forest ecosystem health.

## Introduction

Marine microbes (Bacteria and Archaea) are numerous and diverse, with an estimated richness of a million species globally and abundance of 10^6^ cells per milliliter of seawater (Azam et al., 1983; Whitman et al., 1998). Microbes serve beneficial ecosystem roles, cycling nutrients and providing carbon to higher trophic levels via the microbial loop (Azam et al., 1983; Arrigo, 2005). Microbes further benefit ecosystem function in mediating developmental processes of marine eukaryotes during the critical life stages of recruitment and settlement (Hadfield et al., 2003; Lau et al., 2005; Huggett et al., 2006). As marine microbial communities are susceptible to changes in abiotic environmental conditions, environmental perturbations from anthropogenic activity are exacerbating microbial community shifts (Dinsdale et al., 2008; Haas et al., 2016), which has the potential to feedback to the marine environment and associated macro-organisms.

Anthropogenic influences are responsible for eutrophication of marine environments, where increased nutrients, organic pollution, and toxic metal inputs cause coastal ecosystem disruption (Deheyn and Latz, 2006; Halpern et al., 2008; Biggs and D’Anna, 2012; Waterhouse et al., 2012) and community shifts in macro-organisms (Steneck et al., 2002; Connell et al., 2008; Francini-Filho et al., 2013). Coastal anthropogenic disturbances affect ecosystems by altering microbial abundance, diversity, composition, and metabolic function (Kirchman et al., 2007; Dinsdale et al., 2008; Liu et al., 2010; Kelly et al., 2014; Busch et al., 2015). As a consequence of anthropogenic disturbance in conjunction with microbial feedbacks to the environment, the physiology of associated biota and health of coastal ecosystems is deteriorating (Harvell, 1999; Hewson et al., 2014). This phenomenon has been well-documented in coral reef ecosystems; microbial shifts are occurring where beneficial pelagic microbes are being replaced with disease-inducing pathogens, and higher microbial cell abundance, or microbialization, of the environment is becoming more prevalent (McDole et al., 2012; Haas et al., 2016). Given that microbes play a role in successful recruitment and viability in the early life stages of eukaryotic organisms, a change in the function, pathogenicity, structure, or abundance of the microbial community will have a strong selective force on the survival and distribution of the adult populations; however, studies are required to identify if these biotic interactions between microbes and marine recruits are occurring.

While coral reef ecosystems are recognized to be heavily impacted by microbialization (Haas et al., 2016), kelp forest systems are more resilient to drastic changes due to coastal upwelling regimes that feed the system (Dayton et al., 1984; Foster and Schiel, 1985). However, kelp beds are being subjected to increasingly stressful conditions under climate change, as a disruption in upwelling currents is causing an increase in water temperature and a decrease in nutrient availability. This, in conjunction with more frequent and intense El Niño Southern Oscillation (ENSO) events and trophic cascade disturbances, has thinned kelp forest cover (Vásquez et al., 2006; Raybaud et al., 2013) and kelps are failing to recover from deforestation (Steneck et al., 2002; Foster and Schiel, 2010). The stressful abiotic conditions imposed on kelps makes them more susceptible to disease outbreaks, a relevant concern as warming water temperatures encourages an increase in microbial abundance and prevalence of microbial pathogens in coastal kelp beds (Fernandes et al., 2011; Harder et al., 2012; Lachnit et al., 2013). While the processes regulating kelp forest ecosystems are discussed in terms of top down versus bottom up regulation, there is limited information on the influence of microbial mediation of these interactions, particularly when discussing early life stages that have different selective pressure from adults.

Kelps rely on a heteromorphic alternation of generations to maintain adult populations (Neushul, 1963) which includes microscopic stages. The development of *Macrocystis pyrifera* microscopic zoospores and gametophytes are susceptible to abiotic and biotic conditions in the water column, where propagule release and transit to the recruitment location occur (Deysher and Dean, 1986; Reed et al., 1992; Agrawal, 2009). Kelp zoospore settlement and germination success is impeded under exposure to abiotic environmental extremes, including elevated organic and inorganic nutrient concentrations (Amsler and Neushul, 1989), high salinity levels (Buschmann et al., 2004), increased water temperature, and ultraviolet (UV) radiation (Reed et al., 1992; Graham, 1996; Cie and Edwards, 2008). Few studies to date have described the microbial influence on macroalgal recruitment, which could occur by interaction at three locations, (1) from the algal surface microbiome as the zoospores are released, (2) with the water column microbes as the zoospores transit to the recruitment surface, and (3) with the surface biofilm and water column microbes as the propagules grow. The few studies of the interactions between microbes and recruitment of green and red algae have found mixed results where microbes facilitate and inhibit macroalgal recruitment (Agrawal, 2009; Singh and Reddy, 2014). Natural microbial biofilms promote higher zoospore settlement rates of the green alga *Enteromorpha* sp., compared to sterilized surfaces (Dillon et al., 1989; Joint et al., 2000). The increase in recruitment success under microbial presence was associated with the production of quorum signaling molecules (Patel et al., 2003). Acylated homoserine lactone molecules produced by microbes regulated spore release of red algae *Acrochaetium* sp. (Weinberger et al., 2007), and influenced zoospore settlement of green algae *Ulva* (Tait et al., 2005; Joint et al., 2007) through chemotactic or chemokinetic responses (Joint et al., 2002). Microbial communities are influencing the reproduction and recruitment of the genera of green and red macroalgal groups, and in this study we investigate the effects on brown algae recruits.

We identified the effect of microbes from a nearshore and offshore kelp forest on recruitment of the foundational brown macroalgal species, giant kelp *M. pyrifera*. *M. pyrifera* dominates kelp forest ecosystems along the temperate west coasts of North and South America, the southern coasts of Africa, Australia, and New Zealand. *M. pyrifera* creates complex habitat structures which support diverse and productive ecosystems (Dayton, 1985; Foster and Schiel, 1985; Delille and Perret, 1991; Graham et al., 2007). While *M. pyrifera* is widespread, juvenile sporophytes demonstrate ecotype differentiation, where isolated populations are adapted to survive under a narrow range of environmental conditions in their endemic habitat (Kopczak et al., 1991). We explore kelp propagule–microbial interactions by investigating how the water column microbes affect the success of gametophyte recruitment and growth, by (a) removing microbes, (b) altering microbial community composition, and (c) comparing the effects of microbial communities from nearshore versus offshore environments. We demonstrate from our replicated laboratory recruitment experiments that the collective pelagic microbial community of kelp forests is influencing the recruitment success of giant kelp, *M. pyrifera*.

## Materials and Methods

### Seawater Collection and Microbial Abundance Alteration

Replicate kelp propagule experiments were conducted over summer 2013 and 2014, periods that coincided with the peak kelp recruitment season. Experiments were designed to test the effects of: (a) microbial presence, (b) microbial composition, and (c) microbial abundance on kelp propagule germination and development. Each experiment lasted for approximately 4 weeks. For each experiment, 60 lt of seawater was initially collected and manipulated by microbial treatment, then frozen and thawed for the water changes that occurred throughout each experiment.

For the first experiment, we ran two trials where seawater was collected from the surface of the kelp forest of Point Loma, California (32°39′59.56′′ N, 117°14′50.62′′ W) on 12 August 2013 and 5 September 2013. The water was processed through a 2.00 μm filter that removed eukaryotes which may graze on the spores. In the first treatment the microbial abundance was altered by filtering seawater through a 0.02 μm pore nylon filter, and in the second treatment group the microbes were maintained at environmental abundance with no additional filtering. The seawater collected on 12 August 2013 was used throughout the whole experiment until the end date of 9 September 2013, and remained frozen in between water changes to prevent changes in microbial abundance. Microbial abundance in the seawater was enumerated during weekly water changes. This process was repeated for trial 2 with the 5 September 2013 sample and the experiment ran until 3 October 2013.

The second experiment was designed to test the effects of altered microbial community composition on kelp recruitment. Seawater was collected 5 September 2013 from the Point Loma kelp forest. Microbial abundance and community composition was altered in seawater with broad-spectrum antibiotics rather than filtering. Seawater was first filtered with a 2.00 μm filter to remove eukaryotic grazers, and was treated with (1) ampicillin, (2) erythromycin, (3) kanamycin, (4) streptomycin (50 μg ml^-1^), and (5) a control group without antibiotics.

For the third experiment, the effect of a coastal and offshore microbial community on kelp propagules was tested, by collecting surface seawater from both the kelp forests of Point Loma and Catalina Island, California (33°27′1.89′′ N, 118°29′12.32′′ W) on 28 March 2014 and 11 April 2014, respectively. Point Loma and Catalina Island, CA were selected as collection sites for the investigation due to their contrasting anthropogenic influence. The Point Loma kelp forest is adjacent to San Diego, the eighth most populated city in the United States, and is directly west of the San Diego Bay where there are high levels of commercial, military and recreational boating activities which have resulted in a heavily impacted coastal zone (Halpern et al., 2009; Busch et al., 2015). The kelp forest at Catalina Island is situated 40 km off the coast of California, and the sample location was near a marine protected area adjacent to the University of Southern California Wrigley Marine Science Center with an approximate population of 100 people. Catalina Island is a near-pristine offshore location within the Channel Islands (Halpern et al., 2009). During water collection, environmental water quality measurements, including temperature, salinity, pH, dissolved oxygen, and chlorophyll-*a* were logged at the surface of both collection sites with a MANTA-2 multiprobe (Measurement Specialties, Hampton, VA, USA) (**Table 1**). For both locations, there were four abundance treatments: (1) low, (2) intermediate, (3) environmental, and (4) high. Seawater for all four treatments was initially filtered through a 2.00 μm mesh filter to remove larger eukaryotes that could graze on the spores. Microbial density was adjusted by tangential flow filtration (TFF) to remove microbial cells from the filtrate water fraction (100 kDa pore size), while increasing microbial cell abundance in the retentate fraction (Haas et al., 2014). Seawater for the ‘low’ treatment was collected from the TFF filtrate where microbes were removed. Water for the ‘environmental’ treatment was obtained from the 2.00 μm filtered fraction prior to TFF and microbial abundance was unaltered from seawater collected in the field. The ‘intermediate’ treatment was a mixture of 50% ‘low’ and 50% ‘environmental’ seawater. Seawater for the ‘high’ treatment was collected from the TFF retentate fraction where microbes suspended in 10 lt of seawater were condensed to 1 lt of seawater.

**Table 1:** Water quality measurements logged with a MANTA-2 multiprobe for the Point Loma and Catalina Island, California kelp forests.

	Depth (m)	Temp (°C)	Salinity (ppt)	pH	Chlorophyll-*a* (mg l^-1^)	Dissolved oxygen (% Sat)	Dissolved oxygen (mg l^-1^)
Point Loma	0.607 ± 0.047	16.807 ± 0.039	40.308 ± 0.039	8.073 ± 0.055	1.347 ± 0.015	118.067 ± 2.034	9.367 ± 0.153
Catalina Island	0.230 ± 0.006	16.593 ± 0.009	40.205 ± 0.010	8.847 ± 0.009	1.253 ± 0.018	115.000 ± 0.808	9.177 ± 0.064

*Values displayed are the mean for three measurements taken within 0.50 m, with standard error.*

### Microbial Enumeration

Microbial abundance was quantified throughout the experiments during each weekly water change with colony forming unit counts (CFU) on MacConkey, marine, and TCBS agar. In addition, microbial abundance of the experimental seawater was quantified at the beginning and end of the experiment with epifluorescence microscopy. In short, microbial cells were fixed with paraformaldehyde and collected on a 0.02 μm Anodisc filter (Whatman, UK) and stained with SYBR green. Filters were mounted on slides and stored at -20°C. Cell abundance was counted in replicate using ImagePro Software (Media Cybernetics, Rockville, MD, USA) for cell size range 0.20–10.00 μm (McDole et al., 2012; Haas et al., 2014).

### Spore Release

*Macrocystis pyrifera* reproductive sporophylls with visible fertile sori were collected 12 August 2013 (experiment 1, trial 1), 05 September 2013 (experiment 1, trial 2 and experiment 2), and 28 March 2014 (experiment 3) from the Point Loma kelp forest at the same time and location where seawater was collected. Sporophylls were prepared for laboratory spore release in a protocol modified from Carney and Edwards (2010). Briefly, sporophylls were transported back to the laboratory in a dark cooler within 2 hrs, rinsed with 0.02 μm filtered seawater (FSW), and wiped with paper towels to dislodge epiphytes and reduce the microbial biofilm. Rinsed sporophylls were layered between damp paper towels and desiccated for 8 h at 4°C. A spore release was induced by placing desiccated sporophylls in room temperature 0.02 μm FSW until visual confirmation as indicated by clouded seawater. Spore density was calculated using a hemacytometer under a compound microscope and approximately 500 μl of seawater containing spores were added to each Petri dish. Sporophylls were collected from multiple individuals and spores were pooled to reduce variation in survival by different individuals. Triplicate microcosms were set up for each treatment group across all three experiments, and zoospores for each treatment came from the same pool.

### Experimental Design and Measurements

**FIGURE 1:**
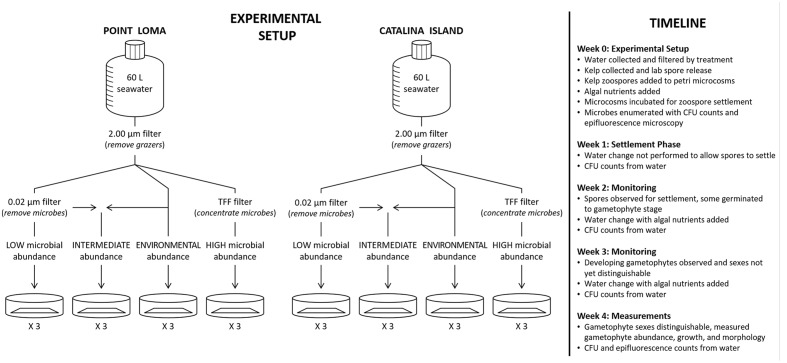
**A diagram of our experimental design testing the effects of altered abundance of microbes from a nearshore (Point Loma) versus an offshore (Catalina Island) kelp forest on kelp recruitment success.** Seawater was collected from the environment and filtered according to abundance treatment – low, intermediate, environmental, and high. Kelp zoospores and gametophytes were monitored weekly for development.

Within all treatment groups for the three experiments – (a) microbial presence, (b) microbial composition, and (c) microbial abundance – the design was as follows. Appropriately manipulated seawater for each microbial treatment group was added to Petri dish microcosms at a volume of 25 ml. *M. pyrifera* haploid swimming zoospores were added to Petri dish microcosms containing seawater at a target density of 50 spores mm^-2^ to reduce density-dependent settlement effects. Zoospores were allowed to incubate in Petri dishes for 2 weeks following spore release without water changes or additional nutrient addition, so as not to disrupt zoospore settlement. Water was changed at 2 weeks post-spore release, and every week thereafter. During water changes, autoclaved Alga-Gro (Carolina Biological, Burlington, NC, USA) growth medium was added to induce kelp propagule development. Petri dish microcosms were incubated (Percival Scientific, Inc., Perry, IA, USA) at 12°C, 12:12 diurnal cycle, and irradiance level of 20 μmol photons m^-2^ s^-1^ (Cie and Edwards, 2008). Microcosms were monitored weekly to check for zoospore germination to gametophyte and gametophyte growth, and images from three haphazardly-selected fields of view in each Petri dish were taken weekly with a Leica digital camera (Leica Microsystems, Bannockburn, IL, USA) attached to an inverted microscope (Leitz Wetzlar, McBain Instruments, Simi Valley, CA, USA). Gametophyte abundance, length, and male:female sex ratios were measured in each field of view at 28 days post-spore release as gametophytes were developed enough to distinguish sex (5.65 mm^2^, 4X magnification) using ImageJ software (National Institutes of Health, Bethesda, MD, USA) (**Figure 1**).

Two-sample, two-sided *t*-test compared mean gametophyte abundances and length between filtered treatments. One-way analyses of variance (ANOVA) were used to compare mean gametophyte abundance and length in antibiotic, Point Loma, and Catalina seawater treatments. Kruskal–Wallis ANOVAs were used when unequal variance was detected. *Post hoc* pairwise multiple comparison procedures (Tukey tests) were used to detect significant differences in mean gametophyte abundance and length. Dunn’s method for pairwise multiple comparisons was substituted for Tukey tests when unequal variance was detected.

## Results

### Microbial Presence and Kelp Recruitment

**FIGURE 2:**
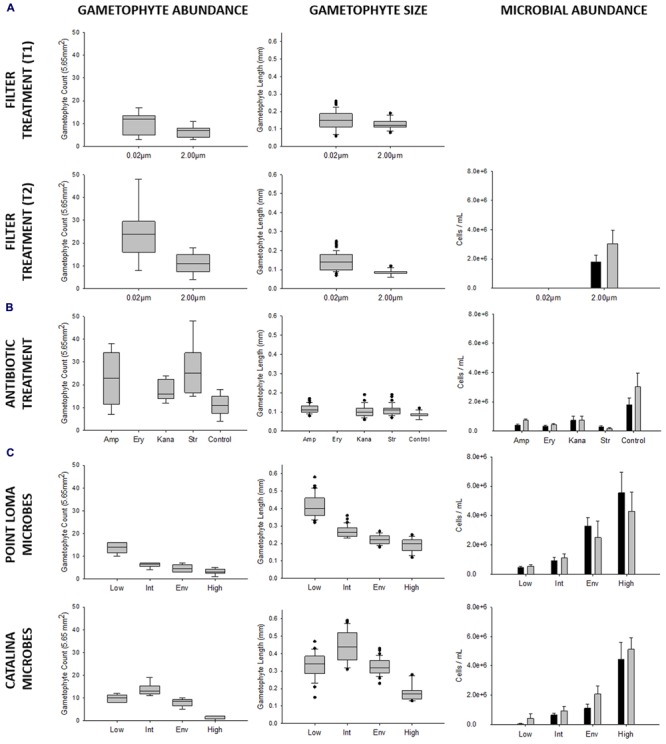
**Results from experiments investigating effects of microbial abundance and community composition on *Macrocystis pyrifera* zoospore settlement and gametophyte development, conducted over the 2013 and 2014 kelp recruitment seasons.** In repeated experiments we tested **(A)** microbial presence, **(B)** microbial composition (antibiotic treatment: amp = ampicillin, ery = erythromycin, kana = kanamycin, str = streptomycin, control = no antibiotics), and **(C)** microbial abundance (Point Loma: low = 4.683 × 10^5^, intermediate = 9.251 × 10^5^, environmental = 3.296 × 10^6^, high = 5.550 × 10^6^; Catalina: low = 0.463 × 10^5^, intermediate = 6.504 × 10^5^, environmental = 1.110 × 10^6^, high = 4.460 × 10^6^) on kelp propagule success. Propagule germination was measured by gametophyte abundance, and propagule development was measured by gametophyte size. *M. pyrifera* gametophyte abundance was recorded per field of view (5.65 mm^2^). Microbial cell counts (ml^-1^) were enumerated with epifluorescence microscopy (cells ml^-1^) at the beginning and end of the experiment as indicated by black and gray bars, respectively (0.20–10.00 μm cell size range).

To first describe the interaction between a coastal microbial community and kelp recruitment, we reared kelp propagules in laboratory experiments in the presence and absence of microbes. We conducted two experimental trials where we reduced microbial abundance by filtering (0.02 μm filter) or maintained microbes at their environmental abundance (Trials 1 and 2). Trials were not combined in analysis, due to a potential variation in kelp genotype and zoospore settlement density. Mean gametophyte abundance was higher in treatments where microbes were removed by 0.02 μm filtering (T_1_: *x* = 10.22 ± 1.60; T_2_: *x* = 24.00 ± 3.87 gametophytes), compared to treatments where microbes were maintained (T_1_: *x* = 6.33 ± 0.87; T_2_: *x* = 11.22 ± 1.52 gametophytes) (T_1_: *t*_df__=__16_ = 2.14, *p* = 0.048; T_2_: *t*_df__=__16_ = 3.07, *p* = 0.007) (**Figure 2**; **Table 2**). Size of *M. pyrifera* gametophytes was affected by microbial abundance, with increased mean gametophyte size with reduced number of microbes (T_1_: *x* = 0.152 ± 0.009; T_2_: *x* = 0.143 ± 0.005 mm) compared to treatments where microbes remained at environmental levels (T_1_: *x* = 0.126 ± 0.006; T_2_: *x* = 0.086 ± 0.002 mm) (T_1_: *t*_df__=__60_ = 2.31, *p* = 0.024; T_2_: *t*_df__=__158_ = 10.32, *p* < 0.001) (**Figure 2**; **Table 2**). Proportion of male:female gametophytes did not change across treatments (*p* = 0.214).

**Table 2:** Multiple statistical comparisons of the gametophyte abundance and size (mm) in experiments investigating the effects of altered microbial abundance and community composition on *Macrocystis pyrifera* recruitment.

	Gametophyte abundance				Gametophyte length			
	Comparison	Differences of means/rank	t/q/Q	*p* < 0.05	Comparison	Differences of means/rank	t/q/Q	*p* < 0.05
Filter experiment (T1)	Filtered versus Non-filtered	3.89	2.14	Yes	Filtered versus Non-filtered	0.03	2.31	Yes
Filter experiment (T2)	Filtered versus Non-filtered	15.67	3.59	Yes	Filtered versus Non-filtered	0.06	10.73	Yes
Antibiotic experiment	Str versus Control	15.22	5.35	Yes	Amp versus Control	93.34	6.61	Yes
	Str versus Kana	8.89	3.13	*No*	Amp versus Kana	46.43	3.04	Yes
	Str versus Amp	3.33	1.17	*No*	Amp versus Str	14.63	1.07	*No*
	Amp versus Control	11.89	4.18	Yes	Str versus Control	78.71	6.50	Yes
	Amp versus Kana	5.56	1.95	*No*	Str versus Kana	31.79	2.36	*No*
	Kana versus Control	6.33	2.23	*No*	Kana versus Control	46.91	3.38	Yes
Point Loma microbes	Low versus High	10.50	15.06	Yes	Low versus High	97.00	10.63	Yes
	Low versus Env	9.00	12.91	Yes	Low versus Env	74.75	6.55	Yes
	Low versus Int	7.50	10.76	Yes	Low versus Int	41.32	3.83	Yes
	Int versus High	3.00	4.30	Yes	Int versus High	55.67	5.50	Yes
	Int versus Env	1.50	2.15	*No*	Int versus Env	33.42	2.73	Yes
	Env versus High	1.50	2.15	*No*	Env versus High	22.24	2.06	*No*
Catalina microbes	Int versus High	12.17	15.53	Yes	Int versus High	99.04	7.16	Yes
	Int versus Env	5.67	7.23	Yes	Int versus Env	47.08	5.39	Yes
	Int versus Low	3.83	4.89	Yes	Int versus Low	43.75	4.79	Yes
	Low versus High	8.33	10.64	Yes	Low versus High	55.29	4.04	Yes
	Low versus Env	1.83	2.34	*No*	Low versus Env	3.33	0.39	*No*
	Env versus High	6.50	8.30	Yes	Env versus High	51.96	3.88	Yes

*Treatments within the antibiotic experiment are defined as: amp = ampicillin, kana = kanamycin, str = streptomycin, control = no antibiotics. The erythromycin treatment was removed from this analysis. Treatments within the Point Loma microbes experiment are defined as: low = 4.683 × 10^*5*^, intermediate = 9.251 × 10^*5*^, environmental = 3.296 × 10^*6*^, high = 5.550 × 10^*6*^ cells ml^*-1*^. Treatments within the Catalina microbial experiment are defined as: low = 0.463 × 10^*5*^, intermediate = 6.504 × 10^*5*^, environmental = 1.110 × 10^*6*^, high = 4.460 × 10^*6*^ cells ml^-*1*^. Gametophyte abundance was recorded in each field of view (5.65 mm^2^). Kruskal–Wallis one way ANOVAs were used when unequal variance was detected. *Post hoc* pairwise multiple comparison procedures (Tukey tests) were used to detect significant differences in mean gametophyte abundance and length. Dunn’s method for pairwise multiple comparisons was substituted for Tukey tests when unequal variance was detected.*

### Microbial Composition and Kelp Recruitment

Mean gametophyte abundance and size differed among antibiotic treatments (ampicillin, erythromycin, kanamycin, streptomycin, control). Erythromycin had an adverse effect on *M. pyrifera* zoospores, as gametophytes failed to appear in all replicates (**Figure 2**) and was removed from further analysis. Gametophytes were significantly different in abundance (*F*_df__=__3_ = 5.50, *p* = 0.004) and size (*H*_df__=__3_ = 60.21, *p* < 0.001) between the antibiotic and control treatments. Streptomycin- and ampicillin-altered microbial communities increased gametophyte abundance (*x*_amp_ = 23.11 ± 3.85; *x*_str_ = 26.44 ± 3.61) compared to the control group (*x* = 11.22 ± 1.52), and all three antibiotics resulted in larger gametophytes (*x*_amp_ = 0.115 ± 0.003; *x*_kana_ = 0.102 ± 0.004; *x*_str_ = 0.111 ± 0.003 mm) compared to the control.

### Nearshore versus Offshore Microbial Communities on Gametophyte Development

**FIGURE 3:**
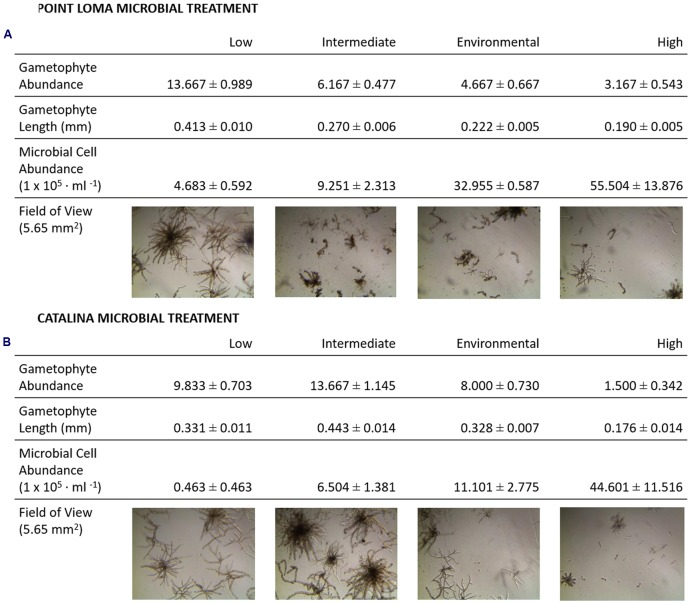
**Results from experiments investigating effects of microbial abundance and community composition on *M. pyrifera* recruitment.** In addition to quantifying the kelp propagule success with gametophyte measurements, a qualitative analysis was performed on images of developing *M. pyrifera* gametophytes. Morphological development of gametophytes were assessed under low, intermediate, environmental, and high microbial abundances for the **(A)** Point Loma and **(B)** Catalina Island microbial treatments. Microbial abundance counts are presented from epifluorescence microscopy data collected at the beginning of the experiment (cells ml^-1^).

The third experiment directly compared the effects of the contrasting Point Loma and Catalina microbial communities on *M. pyrifera* propagules. In agreement with experimental results from the first four trials conducted in the previous recruitment season, as microbial abundance increased in Point Loma seawater, gametophyte abundance decreased (*F*_df__=__3_ = 44.74, *p* < 0.001) (**Table 2**; **Figure 3**) and size decreased (*H*_df__=__3_ = 120.83, *p* < 0.001). When microbial abundance was low (4.683 × 10^5^ cells ml^-1^), gametophyte abundance (*x* = 13.67 ± 0.99) and size (*x* = 0.413 ± 0.010 mm) were significantly higher compared to intermediate (*x* = 6.17 ± 0.48 gametophytes, 0.270 ± 0.006 mm) (9.251 × 10^5^ cells ml^-1^), environmental (*x* = 4.67 ± 0.67 gametophytes, 0.222 ± 0.005 mm) (3.296 × 10^6^ cells ml^-1^), and high (*x* = 3.17 ± 0.54 gametophytes, *x* = 0.190 ± 0.005 mm) (5.550 × 10^6^ cells ml^-1^) microbial abundance treatments. Gametophytes had a deteriorated morphology in the high microbial treatment (**Figure 3**).

Altering microbial abundance in Catalina seawater affected *M. pyrifera* mean gametophyte abundance and size, yet unique trends were identified in contrast to Point Loma treatments. Significant differences were detected in gametophyte abundance (*F*_df__=__3_ = 42.07, *p* < 0.001) and gametophyte length (*H*_df__=__3_ = 61.69, *p* < 0.001) across microbial abundance treatments in Catalina seawater (**Figure 2**). When Catalina microbial abundance was reduced to low (0.463 × 10^5^ cells ml^-1^), mean gametophyte abundance (*x* = 9.83 ± 0.70) and size (*x* = 0.331 ± 0.011 mm) decreased compared to the intermediate (6.504 × 10^5^ cells ml^-1^) microbial abundance treatment (*x* = 13.67 ± 1.15 gametophytes, *x* = 0.443 ± 0.014 mm), but was not significantly different from the environmental (1.110 × 10^6^ cells ml^-1^) microbial abundance treatment (*x* = 8.00 ± 0.73 gametophytes, *x* = 0.328 ± 0.007 mm). Increasing microbial abundance in Catalina seawater to high (4.460 × 10^6^ cells ml^-1^) reduced gametophyte abundance (*x* = 1.50 ± 0.34) and size (*x* = 0.18 ± 0.014 mm) compared to the low, intermediate, and environmental treatments (**Table 2**; **Figure 2**). The morphology of gametophytes was normal until the high treatments where gametophyte development was stunted (**Figure 3**).

Water quality measurements from the Point Loma and Catalina Island kelp forests were similar for dissolved oxygen (*p* = 0.345) and salinity (*p* = 0.107). In Point Loma, temperature was higher (*p* = 0.027), chlorophyll-*a* was higher (*p* = 0.016), and pH was lower (*p* = 0.004) compared to Catalina (**Table 1**).

## Discussion

Our three recruitment experiments showed that the microbial community from the coastal kelp forest of Point Loma negatively affected the recruitment of kelp. We showed this effect three independent times, first with a reduction in microbes by filtering over two replicated trials. The removal of microbes from the water increased the number of kelp propagules and increased their rate of growth (**Figure 2**). Apart from altering microbial abundance, all other water quality parameters remained unchanged in both the filtered and non-filtered microcosms. The filtering treatment was successful in removing microbes and there was no increase in microbial counts during the experiment via introduction with the spores or water changes (**Figure 2**).

We selectively altered the Point Loma microbial community composition with antibiotics, as a method to eliminate disease-inducing pathogens from the seawater. Some broad-spectrum antibiotics, such as ampicillin, were used to reduce abundance of many Gram-positive and Gram-negative bacterial genera including *Streptococcus*, *Staphylococcus*, and *Enterococci*. More selective antibiotics, such as streptomycin, were used to reduce specific bacterial species including those similar to *Mycobacterium* spp. and *Staphylococcus* spp. Although, we did not anticipate testing the direct effects of antibiotics on kelp recruitment, our study found erythromycin to be detrimental to kelp development. Erythromycin is effective against bacterial genera *Streptococcus*, *Staphylococcus*, and *Haemophilus*, and also interferes with photosynthetic processes and cell wall integrity in plants (Agrawal, 2009) and similarly affected the kelp. The addition of the three types of antibiotics halved the number of microbes present and increased the number of propagules that settled compared to the unchanged control group (**Figure 2**), but there was no consistent pattern among treatment groups. This suggests that it was not the reduction in a specific pathogen, but a reduction in the numbers of microbes that affected gametophyte abundance and development. With the exception of erythromycin, the other three antibiotics had no apparent negative effect on the kelp, as all recruits were more numerous and grew larger in the presence of the antibiotic treatment.

Our last experimental recruitment trial was conducted to identify potential differential effects of pelagic microbial communities from a nearshore, anthropogenically-influenced kelp forest (Point Loma, CA, USA) versus an offshore kelp forest (Catalina Island, CA, USA) on *M. pyrifera* propagules. Pelagic microbial communities in the kelp forest will come into contact with kelp propagules upon zoospore release, while zoospores germinate into gametophytes, and while gametophytes develop, and we aimed to test the microbial interaction at these phases in recruitment. We found that low microbial abundance was optimal for kelp recruitment in the Point Loma treatments, yet Catalina microbial treatments showed enhanced kelp recruitment and gametophyte growth at intermediate microbial abundance (**Figure 3**). Point Loma pelagic microbes are inhibitory to kelp recruitment as shown on three occasions, whereas Catalina microbes are facilitating recruitment. Kelps are adapted to a specific range of abiotic conditions in their endemic habitat (Kopczak et al., 1991), and may also be acclimated to their associated microbiota. However, we did not detect signals of an increased recruitment success of Point Loma kelp propagules in Point Loma microbial treatments compared to the Catalina microbial treatment. Overall, our experimental results show that *M. pyrifera* zoospore settlement, gametophyte development, and morphological development are all affected by pelagic microbial presence, and the effects of microbes need to be included when investigating the effects other environment variables on kelp recruitment success.

Our quantification of microbial cell abundance in manipulated seawater treatments suggests that microbial abundance remained consistent from the beginning to the end of the experiment, likely due to freezing the seawater between weekly water changes to suspend microbial proliferation (**Figure 2**; **Supplementary Figure S1**). Because microbial abundance was manipulated directly from the kelp forest seawater, microbes in the experimental groups were representative of environmental microbial communities. Although, nutrients were not quantified in our experimental microcosms, nutrient concentrations for each experimental group would have remained unchanged from the environmental conditions due to the filtering techniques. Therefore, we assert that the microbes, rather than the nutrients, were correlated with the changes observed in kelp recruitment across our three experiments.

The kelp forests of Point Loma and Catalina Island have contrasting anthropogenic influence (Halpern et al., 2009), and we suspect that the microbial taxonomic and functional composition are different and indicative of their respective environmental characteristics. In nutrient-enriched nearshore marine environments, heterotrophic and copiotrophic microbial taxa are present (Haggerty and Dinsdale, 2016). Heterotrophic and copiotrophic taxa break down complex organic matter, metal contaminants, and participate in the carbon and nitrogen cycles in nutrient-enriched environments (Selje et al., 2004; Bauer et al., 2006; Dang et al., 2008). Point Loma, adjacent to the populated city of San Diego, has elevated nutrient and trace metal input as a result of increased anthropogenic activity (Deheyn and Latz, 2006; Biggs and D’Anna, 2012), which has altered the heavy metal resistance ability of the microbial community within the kelp forest (Busch et al., 2015). A higher level of chlorophyll-*a*, a proxy for nutrient level estimation, was identified at Point Loma compared with Catalina (**Table 1**). Conversely, we expect the Catalina Island microbial community to be characterized by oligotrophic, phototrophic microbes adapted to low nutrient availability (Morris et al., 2002; Haggerty and Dinsdale, 2016), based on the low level of chlorophyll-*a* that we detected (**Table 1**). To understand why the Point Loma and Catalina Island microbial communities were affecting *M. pyrifera* propagule success in a different manner, we suggest that a metagenomics analysis of the water column, kelp surface, and the biofilm are necessary.

As anthropogenic activity increases in coastal environments worldwide, marine microbial communities are changing. Shifts in abundance, taxonomy, and metabolism of microbial communities are affecting the health of associated marine macro-organisms, and prevalence of microbial-induced disease in macro-organisms is increasing (Harvell, 1999; Harvell et al., 2002; Hewson et al., 2014; Haas et al., 2016). Taxonomic changes in microbial communities have occurred with elevated anthropogenic activity on coral atolls of the Line Islands (Dinsdale et al., 2008; McDole et al., 2012; Kelly et al., 2014; Haas et al., 2016) and were correlated with declining coral cover. Microbial-induced diseases are detrimental to algae species, inflicting bleaching disease in red algae (Fernandes et al., 2011), and evident tissue deterioration in southern California *M. pyrifera* populations occurs periodically (Rosenthal et al., 1974). Kelp forests in coastal California showed major declines in the 1960 and 1980s and while some restoration has occurred, kelp forests have not been restored to pre-1960 levels (Foster and Schiel, 1985). Over recent years, increased sea urchin grazing due to trophic cascade disturbances, increased ocean temperature, and more frequent El Niño Southern Oscillation (ENSO) events have steadily diminished *M. pyrifera* populations along the southern California coastline (Edwards and Hernández-Carmona, 2005). Similarly, coastal kelp populations in other metropolitan regions around the world have declined (Johnston et al., 2011; Marzinelli et al., 2013; Campbell et al., 2014). The anthropogenic influence on the microbes and their influence on recruitment that we have described is a potential cause of the decline in kelp. Our study emphasizes how microbial cell abundance and microbial composition affects recruitment of an iconic and foundational coastal macro-organism and may, in time, affect population levels.

Microbes are important players in the health of marine organisms (Azam et al., 1983; DeLong and Pace, 2001), and we have begun to describe the influence of microbes on a key stage of reproduction and growth of a foundational coastal kelp. Kelps like all species rely on successful recruitment to restore populations (Neushul, 1963) and we have shown that the nearshore microbes are detrimental to kelp recruits at high abundances. If recruitment becomes hindered by unfavorable abiotic and biotic conditions, including altered microbial abundance and composition, kelp distribution and abundance will continue to decline. Subsequently, ecosystems reliant on kelp forests for structure, nutrients, protection, and economic benefits will suffer (Graham, 2004). Our study shows that pelagic microbial communities associated with kelp forests near areas of high human activity affects recruitment of *M. pyrifera*, and these results will need to be incorporated into kelp forest ecosystem models.

This is the first study to describe the microbial interactions with recruitment of giant kelp, *M. pyrifera*. Notably, we tested pelagic influence of microbial assemblage on kelp recruits. We have established one interaction between pelagic microbes and released propagules of *M. pyrifera*; yet in accordance with other studies, we postulate that microbes are affecting multiple recruitment processes across other water column and biofilm locations in the kelp forest. As part of an ongoing study of the microbial ecology of kelp forests, we are currently investigating the kelp surface and benthic substrate biofilm microbial communities on kelp reproductive success, with both metagenomic and experimental approaches. With future studies we will improve our understanding of the role of microbes in the complex network of biotic and abiotic interactions on recruitment of giant kelp, *M. pyrifera*. Whether the microbial community surrounding the kelp propagules interacts independently or synergistically with other biotic or abiotic conditions to affect recruitment levels in kelp is an outstanding question. Investigating the combined effects of microbes and environmental variables, such as temperature and pH, on kelp recruitment will identify whether the microbial effects on kelp recruits will be greater under the imminent threats of global climate change.

## Author Contributions

MM, ED, and ME conceived and designed experiments. MM carried out experiments and collected data, MM, JH, BP, and ED performed data analysis. MM, JH, ME, and ED contributed in field collection. AV contributed to microbial enumeration data. MM and ED wrote the first draft of the manuscript, and all authors contributed substantially to revisions.

## Conflict of Interest Statement

The authors declare that the research was conducted in the absence of any commercial or financial relationships that could be construed as a potential conflict of interest.

## Supplementary Material

FIGURE S1 **Microbial abundance was enumerated weekly from the experimental seawater for each treatment testing microbial: (A)** presence or absence, **(B)** composition, and **(C)** abundance on kelp recruitment. Data points indicate the mean colony forming unit (CFU) count for *n* = 3 of each treatment ± SEM..
