# Supplementary figures and images for: Nearshore Pelagic Microbial Community Abundance Affects Recruitment Success of Giant Kelp, Macrocystis pyrifera
Source: Front Microbiol. 2016 Nov 14;7:1800. doi: 10.3389/fmicb.2016.01800 (PMC5107569; doi:10.3389/fmicb.2016.01800)

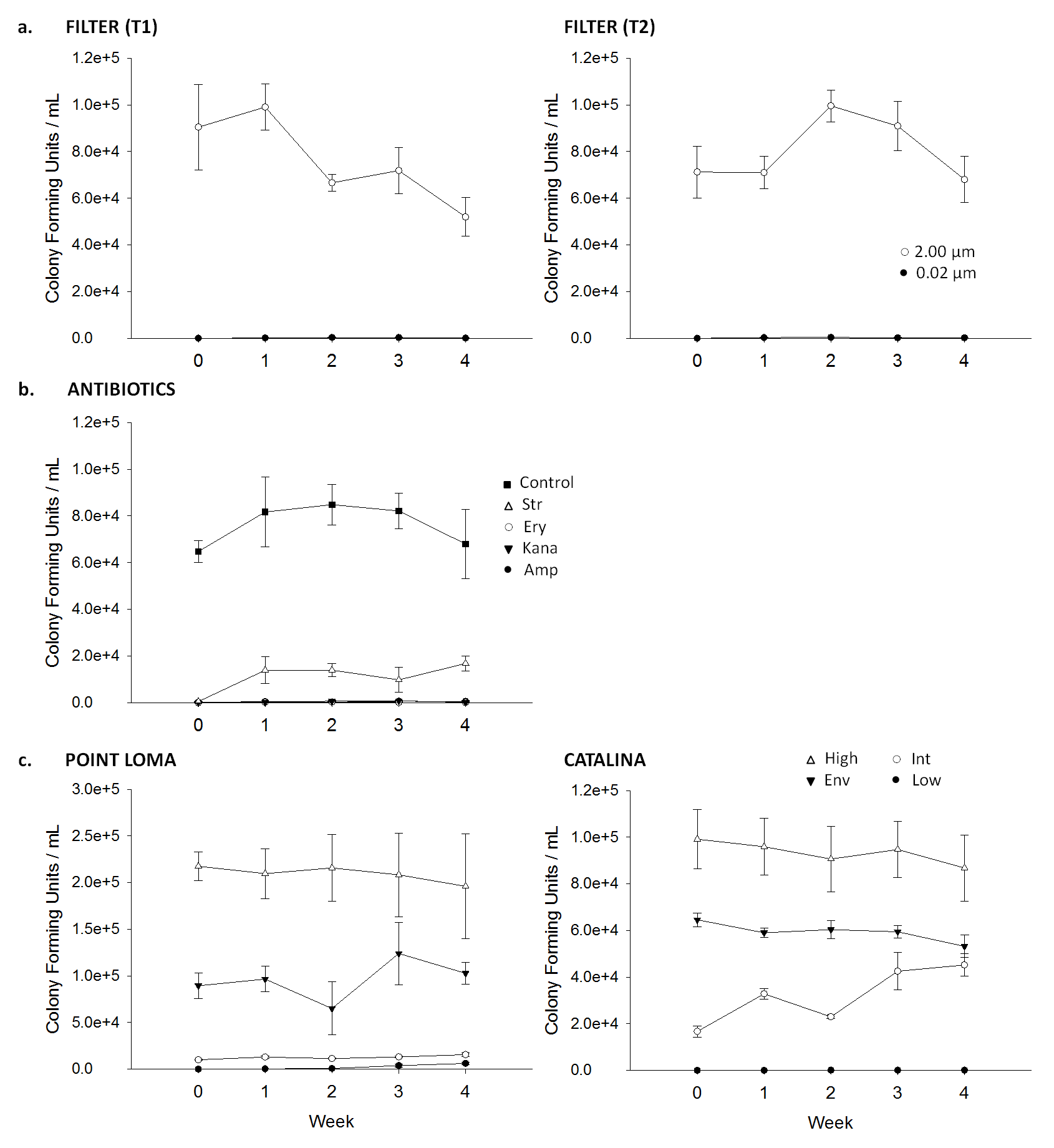

Supplement: FIGURE S1 — Microbial abundance was enumerated weekly from the experimental seawater for each treatment testing microbial: (A) presence or absence, (B) composition, and (C) abundance on kelp recruitment. Data points indicate the mean colony forming unit (CFU) count for n = 3 of each treatment ± SEM.. [file Image_1.TIF]
